# *CVF1* Promotes Invasive *Candida albicans* Infection via Inducing Ferroptosis

**DOI:** 10.3390/jof11050342

**Published:** 2025-04-27

**Authors:** Gang Luo, Yongman Ma, Chunyi Chen, Yudie Hu, Chunchun Yan, Di Wang, Cong Wang, Yanyan Wang, Xichen Yu, Andriy Sibirny, Jun Yuan, Yingqian Kang

**Affiliations:** 1 Key Laboratory of Microbiology and Parasitology of Education Department of Guizhou, School of Basic Medical Science, Guizhou Medical University, Guiyang 561113, China; 2 Department of Epidemiology and Health Statistics, School of Public Health, Guizhou Medical University, Guiyang 561113, China; mayongman1022@163.com; 3 Key Laboratory of Environmental Pollution Monitoring and Disease Control, Ministry of Education, School of Basic Medical Sciences, Guizhou Medical University, Guiyang 561113, China; 4 Institute of Cell Biology, NAS of Ukraine, Drahomanov Street 14/16, 79005 Lviv, Ukraine; sibirny@cellbiol.lviv.ua; 5 Institute of Biotechnology, University of Rzeszow, Zelwerowicza 4, 35-601 Rzeszow, Poland; 6 Department of Laboratory Medicine, Guiyang Second People’s Hospital, Guiyang 550081, China; junyuan99430@163.com

**Keywords:** host–pathogen interactions, invasive fungal infection, virulence factor, ferroptosis

## Abstract

Recent studies have shown that several pathogens manipulate ferroptosis in host cells to aid their dissemination and enhance pathogenicity. While bacterial virulence factors capable of inducing ferroptosis have been identified, no such factors have been reported for human fungal pathogens thus far. *Candida albicans*, a most common human pathogenic fungus causing invasive fungal diseases, has recently been found to be able to induce ferroptosis in macrophages. Whether specific virulence factors induce ferroptosis in host cells that promote *C. albicans* pathogenicity remains to be defined. Here, we identify *CVF1* as a critical virulence gene of *C. albicans* that is required for systemic fungal infection. Moreover, the *CVF1* gene can significantly promote macrophage death. Using a macrophage infection model combined with the addition of cell death inhibitors, we show that the *CVF1*-induced death of macrophages is attributed to ferroptosis. More importantly, *CVF1* is sufficient to trigger ferroptosis to promote *C. albicans* dissemination and pathogenicity in vivo. This study highlights a mechanism by which a virulence factor from a human fungal pathogen regulates ferroptosis in host cells, supporting the concept that human pathogenic fungi harbor specific virulence factors to manipulate ferroptosis in host cells for their invasive infection.

## 1. Introduction

Fungal infections are responsible for the deaths of approximately 1.5 to 2 million individuals globally each year [1]. Among human opportunistic fungal pathogens, *Candida albicans* is the most prevalent, leading to both superficial mucosal infections and life-threatening systemic candidiasis, particularly in immunocompromised individuals [2]. Systemic *C. albicans* infections are especially lethal, contributing to roughly 200,000 deaths annually [3,4]. *Candida*-related infections are one of the 10 most common isolated pathogens in immunocompromised patients or in intensive care units (ICUs) [5], and *Candida* is usually one of the top four causes of bloodstream infections [6]. The immune defense against *C. albicans* is highly dependent on the activity of myeloid cells within the innate immune system [7]. Macrophages, a key type of myeloid phagocyte, are pivotal in both initiating the antifungal immune response and clearing *C. albicans* from infected tissues and the bloodstream [8]. These cells employ specialized strategies to target and eliminate fungal invaders [9].

Regulated cell death (RCD) represents a crucial defense strategy by which host cells combat microbial invaders [10]. Among the various forms of RCD, such as apoptosis, necrosis, and autophagy, these mechanisms are deployed by host cells to curb the spread of infections [11]. Ferroptosis, a more recently discovered form of RCD, is characterized by lipid peroxidation that depends on iron [12]. The process of ferroptosis is modulated through three main stages [13,14]. Initially, two critical factors are required to trigger ferroptosis: the buildup of free iron and the suppression of the antioxidant defense system comprising SLC7A11, GSH, and GPX4. This leads to an “intermediate stage” where lipid peroxidation occurs, primarily involving polyunsaturated phospholipids and the activity of lipoxygenases. In the final stage, lipid peroxidation or byproducts such as 4-hydroxynonenal (4-HNE) and malondialdehyde (MDA) cause the plasma membrane to become porous, leading to cell death [12]. Recent studies indicate that several pathogens manipulate ferroptosis to enhance their dissemination and pathogenicity [13,15].

In pathogenic bacteria, studies have shown that some bacterial infections can trigger ferroptosis in host cells [11]. For instance, Dar HH et al. [16] have found that *Pseudomonas aeruginosa* triggers lipid peroxidation, leading to ferroptosis in human bronchial epithelial cells. Another study has found that *Mycobacterium tuberculosis* can trigger ferroptosis in macrophages, thus promoting dissemination [17]. Although some bacteria have been confirmed to trigger ferroptosis in host cells, there have been very few studies on the molecular mechanism of the interaction between pathogenic bacteria and [11]. So far, investigations have primarily focused on *M. tuberculosis*-induced ferroptosis. Notably, the PtpA effector protein of *M. tuberculosis* has been proven to induce ferroptosis via targeting PRMT6, thus inhibiting GPX4 expression and enhancing the bacterium’s pathogenic effects [18]. Furthermore, Bach1, a transcription factor that represses levels of glutathione and Gpx4, has been documented as a pivotal host factor that regulates *M. tuberculosis*-induced ferroptosis [19].

Recent evidence documents that pathogenic fungal infections can also lead to ferroptosis in host cells [11]. As an illustration, ferroptosis has been documented in rice cells infected by the plant pathogen *Magnaporthe oryzae* [20]. In terms of human pathogenic fungi, *C. albicans* has been recently reported to trigger ferroptosis in macrophages, facilitating inflammation and systemic infection [21]. There is increasing evidence pointing to a link between *Cryptococcus neoformans*-induced meningitis and ferroptosis [11]. However, no research has yet identified specific virulence factors in pathogenic fungi, such as *C. albicans*, that drive ferroptosis in host cells or the molecular mechanisms involved.

In this study, a key virulence gene of *C. albicans*, *CVF1* (*orf19.7455*), was discovered to be indispensable for fungal virulence in a mouse model of systemic infection. Furthermore, the *CVF1* gene can significantly promote the death of macrophages. Through the use of various inhibitors targeting different cell death pathways, including apoptosis, necroptosis, pyroptosis, and ferroptosis, the *CVF1*-induced death of macrophages was attributed to ferroptosis. Importantly, *CVF1* could trigger ferroptosis to enhance *C. albicans* pathogenicity and dissemination in vivo. These discoveries lend credence to the idea that pathogenic fungi targeting humans may employ particular virulence factors to modulate ferroptosis within host cells.

## 2. Materials and Methods

### 2.1. Ethics

All animal-related experimental protocols were reviewed and granted by the Institutional Animal Care and Use Committee of Guizhou Medical University. The procedures adhered strictly to the established ethical guidelines and regulations for animal research.

### 2.2. Fungal Strains and Mice

The fungal strains used are detailed in Table S1. Unless specified otherwise, all strains were maintained in YPD broth (1% yeast extract, 2% peptone, and 2% dextrose) at 30 °C. Specific pathogen-free (SPF) female C57BL/6 and male BALB/c mice were purchased from Huafukang Biotechnology Co. Ltd. (Beijing, China). Among them, the SPF C57BL/6 mice were used to prepare BMDM macrophages, and the SPF BALB/c mice were used for animal infection experiments. These mice were housed in a standard SPF-grade animal facility in the Laboratory Animal Research Center at Guizhou Medical University. The conditions for an SPF-grade animal facility mainly include filtered water, clean air, optimal humidity and temperature levels, and appropriate nutritional feed. When infection or further experiments were required, we strictly followed sterile operating procedures for mouse-related assays and continued to feed and observe the mice using SPF equipment.

### 2.3. Construction of Strains 

The *C. albicans* mutant and complement strains were generated as previously described [22,23]. To create the *CVF1* null mutant strain (*cvf1*), the *LEU2* cassette was amplified from the pSN40 plasmid. Fusion PCR was implemented to generate a product of LEU2 flanked by the 5′ and 3′ regions of *CVF1*, which was then introduced into the SN152 strain. To disrupt the second *CVF1* copy, the *HIS1* cassette was amplified from the pSN52 plasmid, and the fusion PCR product of *HIS1*, flanked by the *CVF1* 5′ and 3′ regions, was utilized for transformation. For constructing the *CVF1*-complemented strain (*cvf1+CVF1*), the entire *CVF1* open reading frame (ORF), along with its upstream region and the *ARG4* cassette from the pSN69 plasmid, was amplified. Using a fusion PCR method, the *CVF1* ORF and downstream flanking regions were amplified and introduced into the *cvf1* mutant to restore gene function. The primers for these procedures are summarized in Table S2.

### 2.4. Mouse Infection Assay

For these experiments, six-week-old male and female BALB/c mice weighing 20–22 g were selected. Mice were infected with 5 × 10^5^ yeast cells from the *C. albicans* strains via the lateral tail veins, which were cultured in YPD at 30 °C until saturation. When this dose of wild-type *C. albicans* cells was used to infect male BALB/c mouse models (day 5 post-infection), the fungal loads in the kidney, liver, and spleen of infected mice were approximately 1.4 × 10^5^, 2.1 × 10^4^, and 1.4 × 10^4^ CFUs/g organ, respectively. To inhibit ferroptosis, some mice received a daily intraperitoneal injection of 10 mg/kg Fer-1, starting 6 h after infection [21]. On day 5 post-infection, tissue homogenates from the kidneys, liver, and spleen were serially diluted and plated on YPD agar. Fungal CFUs were counted after incubation at 30 °C for 48 h. Kidney homogenate supernatants were preserved at −80 °C for further analysis of cytokine levels, GSH, GPX4, 4-HNE, and MDA. The remaining mice were observed three times daily for up to 21 days, and those in a moribund state were euthanized following humane protocols.

### 2.5. Infection of BMDMs with C. albicans

BMDMs were generated as described by Kasper L et al. [24]. *C. albicans* cells were incubated with BMDMs (MOI = 1) at 37 °C and 5% CO_2_ in the presence or absence of Fer-1 (Selleck, TX, USA), RSL3, Ebselen, NAC, Z-VAD, Nec-1s, DSF (MCE, NJ, USA) [21]. After 12 h, the supernatants from BMDMs infected with *C. albicans* strains were adopted to detect the levels of LDH, cytokines, GSH, GPX4, 4-HNE, and MDA. Macrophage lysates were utilized to examine the survival rate of *C. albicans*.

### 2.6. LDH Assays

LDH activity in the supernatants of BMDMs infected with *C. albicans* strains was measured employing an LDH activity assay kit (Solarbio Technology, Beijing, China). Absorbance was tested at 490 nm, and LDH release was calculated as a fold change relative to the uninfected control samples. 

### 2.7. Macrophage-Mediated Killing of C. albicans

The survival of *C. albicans* following co-culture with BMDMs was assessed by CFU counts, following methods previously described [21,25]. *C. albicans* cells grown overnight in YPD were incubated with BMDMs at a 1:1 MOI for 12 h, with or without 10 µM Fer-1. Macrophages were lysed with 0.1% Triton X-100 for 2 min, and serial dilutions were plated on YPD agar to determine candidacidal activity by BMDMs. CFU counts were obtained after incubating plates at 30 °C for 48 h and were compared to the CFU counts of the initial inoculum.

### 2.8. Measurement of Cytokine Production

On day 5 post-infection, the levels of the cytokines TNFα, IL-1β, and IL-6 in the supernatants of homogenized kidneys were determined utilizing ELISA kits (Hangzhou Lianke Biotechnology Co., Ltd., Hangzhou, China). For in vitro co-culture experiments with BMDMs, cell samples were harvested, and the amounts of these cytokines were measured in the same way. In qRT-PCR experiments, the cytokine (TNFα, IL-1β, and IL-6) and GAPDH primer sequences were obtained from the studies of Wang et al. [26] and Wang et al. [27], respectively.

### 2.9. Measurement of GSH, GPX4, 4-HNE and MDA Levels

The supernatant from *C. albicans*-infected kidneys homogenates after 5 days, and macrophages after 12 h, were utilized to detect the content of GSH, GPX4, 4-HNE and MDA. Accordingly, the levels of GSH and GPX4 were assessed using a GSH Assay Kit (Beyotime, Shanghai, China) and a GPX4 ELISA Kit (Shanghai Enzyme-linked Biotechnology, Shanghai, China), respectively. The 4-HNE ELISA Kit (Wuhan Yilairuite Biotechnology Co., Ltd., Wuhan, China) and MDA Content Assay Kit (Beijing Boxbio Science & Technology Co., Ltd., Beijing, China) were employed for level determination, respectively.

### 2.10. Statistical Analysis

All measurements are depicted as the means ± standard deviation (SD) of at least three biological replicates. The differences between groups were examined utilizing an independent-sample *t*-test or one-way analysis of variance with Dunnett’s test. *p*-values < 0.05 were deemed to be statistically significant.

## 3. Results

### 3.1. CVF1 Is Essential for C. albicans Pathogenicity During Systemic Infection

To identify potential new virulence genes of *C. albicans*, we consulted RNA-seq data previously published on the linkage between *C. albicans* and the host. Among them, Amorim-Vaz S et al. [28] conducted a transcriptional analysis of *C*. *albicans* in vivo. In this collection of work on RNA-Seq data, in order to overcome the bottleneck of low pathogen loads in infected tissues, which makes it difficult to detect the full pathogen transcriptome in vivo, the authors enriched the RNA of *C. albicans* from mouse kidneys (16 and 48 h) and *Galleria mellonella* (2 and 24 h) at early and late time points, based on the “SureSelect capture system”, and identified the top 20 genes that were significantly upregulated in both early and late stages of *C. albicans*-infected mice and *G. mellonella*. Among these 20 genes, 14 genes (such as *ECE1*, *HWP1*, *ALS3*, and *UME6*) are currently functionally known, and almost all of them play a key role in the pathogenicity of *C. albicans* [29,30,31]. The functions of the other six genes are still unknown, among which *orf19.7455* has the highest upregulation level (2419- to 3566-fold) during infection in all 20 genes. Therefore, we speculate that *orf19.7455* could play a pivotal role in *C. albicans* pathogenicity. We named this gene *CVF1*, suggesting its involvement in host ferroptosis, as detailed below.

**Figure 1:**
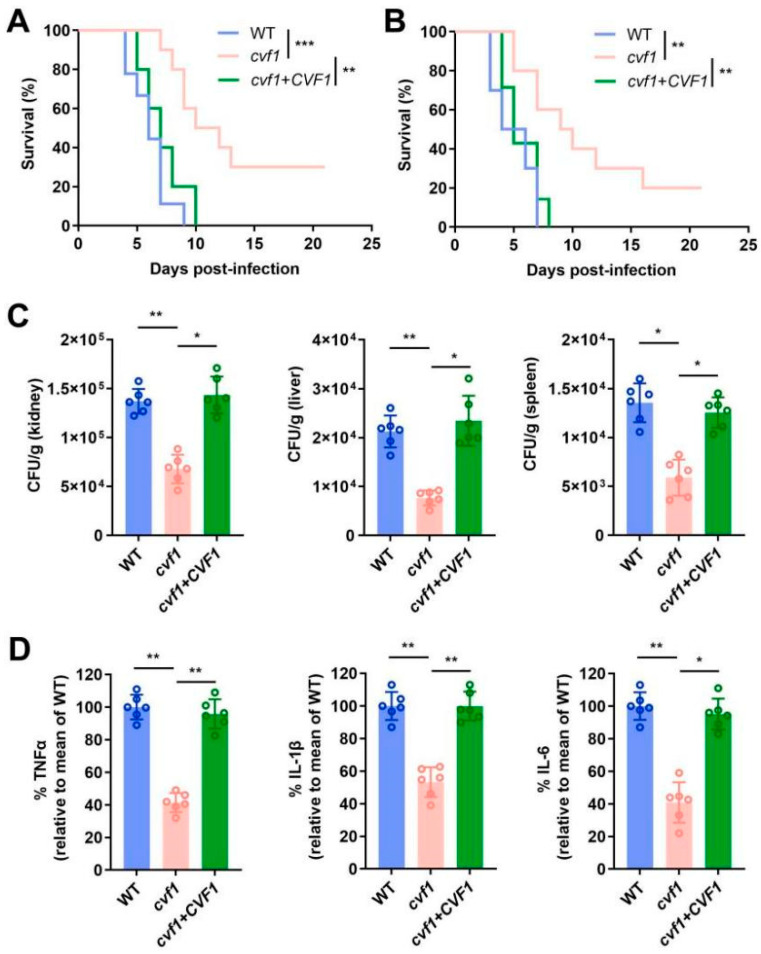
*CVF1* is required for pathogenicity and inflammation. BALB/c mice were infected with 5 × 10^5^ CFU WT, *cvf1*, or *cvf1*+*CVF1* strains via their lateral tail vein. (**A**,**B**) The *cvf1* mutant exhibits reduced lethality in male (**A**) and female (**B**) mice compared to the WT strain or a *cvf1*+*CVF1* gene addback strain. Statistical significance was determined using the Mantel–Cox test. (**C**) Quantification of the fungal burden in the tissues (kidneys, liver, and spleen) of male BALB/c mice infected with indicated *C. albicans* strains at day 5. The results are presented as CFUs per gram of tissue. (**D**) ELISA assays for TNFα, IL-1β, and IL-6 in homogenized kidneys from male BALB/c mice infected with indicated *C. albicans* strains at day 5. The mean of the WT control group was set at 100% to determine the percentage change of TNFα, IL-1β, or IL-6 levels in the mice infected with the *cvf1* or *cvf1*+*CVF1* strains. The data are expressed as means ± SD from six independent biological replicates. * p < 0.05, ** p < 0.01, *** p < 0.001.

We then focused on whether *CVF1* impacted mouse survival. We compared a *cvf1* null mutant strain (constructed in the SN152 background) to both the wild-type (WT) strain and a *cvf1* mutant strain with a reintroduced *CVF1* gene (complemented strain). As depicted in Figure 1A,B, mice infected with the *cvf1* mutant exhibited a notably higher survival rate relative to those with the WT or complemented strains. In contrast, all mice infected with the WT or complemented strains succumbed to the infection within 21 days (Figure 1A,B). Additionally, fungal burden analysis on day 5 post-infection revealed markedly lower fungal loads in the kidneys, liver, and spleen of mice infected with the *cvf1* mutant, as compared to the WT and complemented strains (Figure 1C). The *cvf1* mutant strain exhibited similar hyphal development compared with the WT or complemented strains under in vitro hypha-inducing conditions (Figure S1A,B), and displayed no defect in the growth of the YPD medium (Figure S1C). These findings indicate that *CVF1* is critical in the virulence of *Candida* during invasive infections.

To quantify the degree of inflammation, the levels of the proinflammatory cytokines TNFα, IL-1β, and IL-6 were measured in the kidneys of mice on day 5 after infection. The cytokine levels in mice following infection with the *cvf1* mutant strain were evidently lower than in those infected with the WT or complemented strains (Figure 1D), demonstrating that *CVF1* contributes to the activation of the proinflammatory response in vivo. Collectively, these results uncover that *CVF1* is essential for both virulence and the induction of inflammation during systemic infection.

### 3.2. CVF1 Induces the Death of C. albicans-Infected Macrophages 

**Figure 2:**
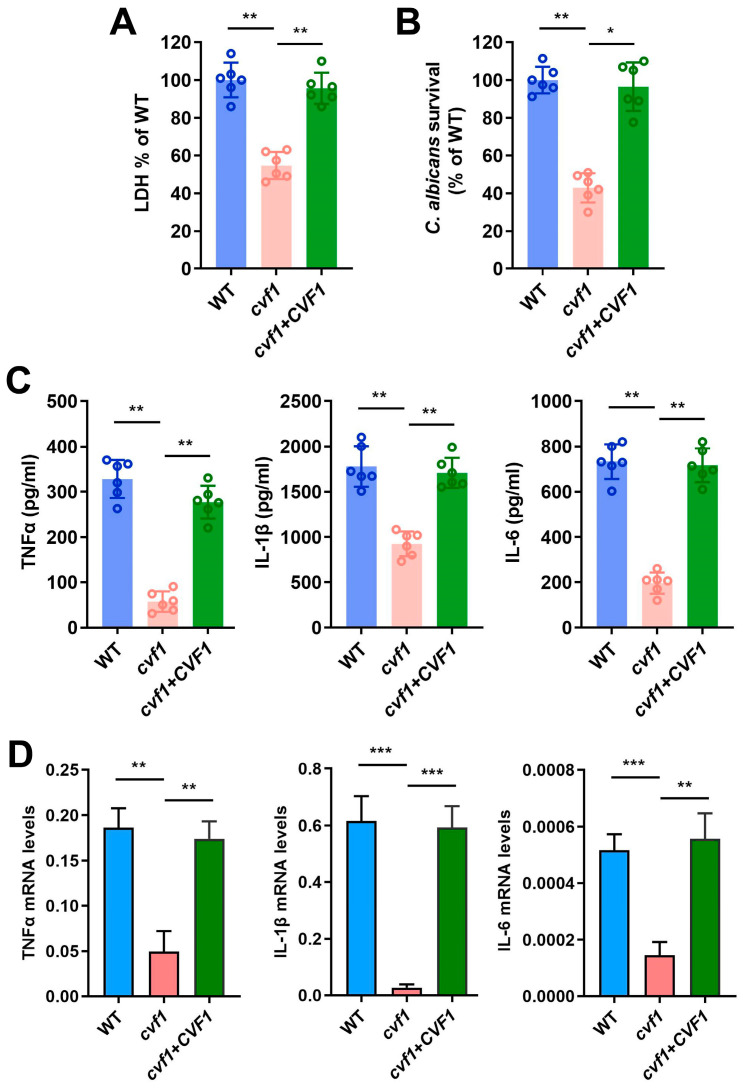
Cells lacking *CVF1* are less efficient in damaging and inducing cytokine responses in BMDMs. BMDMs were infected at an MOI of 1 with WT, *cvf1*, or *cvf1*+*CVF1* strains and incubated at 37 °C and 5% CO_2_ for 12 h. (**A**) Damage of BMDMs was quantified by measuring the release of LDH into the supernatant after co-incubation with indicated *C. albicans* strains. LDH released by the WT was set to 100%. (**B**) Fungal survival was analyzed after co-incubation with BMDMs by CFU plating. The CFUs were recovered and compared to the CFUs in the starting inoculum. Survival of the WT was set to 100%. (**C**,**D**) Expression levels of TNFα, IL-1β, and IL-6 were analyzed using ELISA (**C**) and qRT-PCR (**D**) from macrophages challenged with *C. albicans* WT, *cvf1*, or *cvf1*+*CVF1* strains. qRT-PCR results were normalized to the expression of GAPDH. Data are presented as means ± SD from six biologically independent experiments. * p < 0.05, ** p < 0.01, *** p < 0.001.

Macrophages are crucial for the host’s defense against systemic *C. albicans* infections, so we sought to determine whether *CVF1* plays a role in the pathogen’s cytotoxicity toward macrophages. We hypothesized that *C. albicans* lacking *CVF1* would cause less damage to macrophages. To test this, we assessed macrophage membrane integrity by examining lactate dehydrogenase (LDH) release, a common indicator of cell lysis [32]. Macrophages infected with the *cvf1* mutant strain released distinctly less LDH relative to those with the WT strain (Figure 2A). Moreover, macrophages infected with the complemented strain exhibited similar LDH release to the WT, indicating comparable levels of damage (Figure 2A). Conclusively, *CVF1* is essential for *C. albicans*-induced macrophage lysis.

In order to speculate on the potential molecular mechanism of *CVF1*-promoted pathogenicity, we conducted a bioinformatics analysis of the structure and function of *CVF1*. The results showed that the Cvf1 protein contains a nuclear localization signal, DNA binding regions, and a transcriptional activation regulatory region (Figure S2A). Functional analysis also showed that Cvf1 has potential functions in DNA binding and transcriptional regulation (Figure S2B). Further STRING network analysis showed that Cvf1 could interact with genes such as *TRY5, DAG7*, *CMI1*, *HGT3*, and *ZRT1* (Figure S2C). Among these genes, TRY5 is a Zn(II)2Cys6 transcription factor (www.candidagenome.org). Our recent research has shown that *CMI1* plays a key role in promoting *C. albicans* virulence in mice and macrophages [33], while other genes have not been reported for their virulence functions in mice and macrophages. These results suggest that *CVF1* may promote pathogenicity in the host by entering the nucleus of *C. albicans* and regulating the expression of related virulence genes with a function similar to that of transcription factors.

Given that the *cvf1* mutant causes less macrophage damage, we next investigated whether macrophages could kill the mutant strain more effectively. We measured the survival of *C. albicans* within macrophages, utilizing colony-forming unit (CFU) counts. The *cvf1* mutant exhibited evidently reduced survival compared to the WT and complemented strains (Figure 2B), demonstrating that impaired macrophage damage leads to enhanced clearance of the mutant strain.

Subsequently, we tested whether *CVF1* could be involved in the modulation of the host immune response in macrophages. The WT-infected and complemented-strain-infected macrophages were able to produce a potent cytokine response (Figure 2C,D), while the *cvf1* mutant induced a much lower expression of proinflammatory cytokines in bone marrow-derived macrophages (BMDMs) (Figure 2C,D). Conclusively, *CVF1* contributes to a *C. albicans*-induced immune response in macrophages.

### 3.3. CVF1-Induced Macrophage Death Correlates with a Peroxidized Lipid Signature

**Figure 3:**
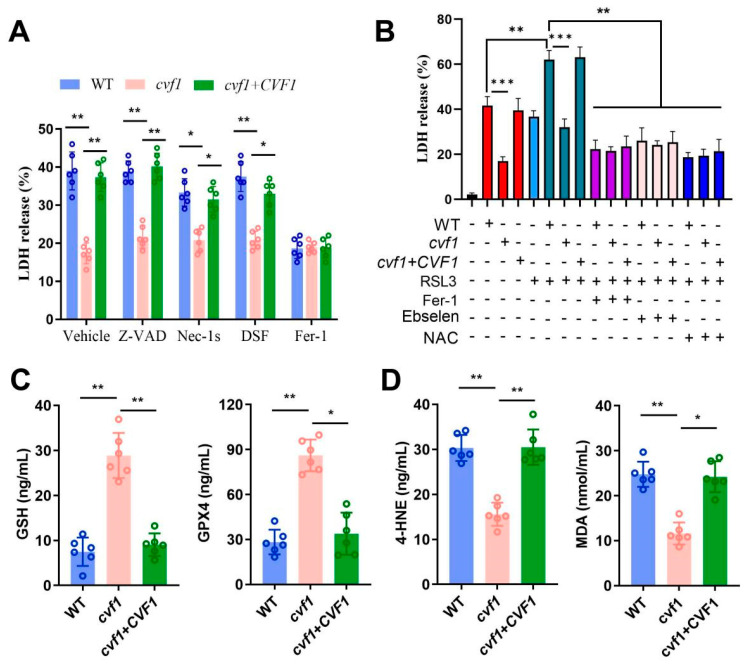
The cell death of *CVF1*-dependent macrophages is associated with a peroxidized lipid signature. BMDMs were infected (MOI 1) with WT, *cvf1*, or *cvf1*+*CVF1* strains and incubated at 37  °C and 5% CO_2_. (**A**) Measurements of LDH release in BMDMs infected with indicated *C. albicans* strains in the presence of z-VAD-FMK (Z-VAD, 1 μM), necrostatin-1s (Nec-1s, 40 μM), disulfiram (DSF, 10 μM), or ferrostatin-1 (Fer-1, 10 μM) for 12 h. (**B**) LDH release of BMDMs infected with indicated *C. albicans* strains after the treatment of ras selective lethal 3 (RSL3, 1 μM), with or without Fer-1 (10 μM), Ebselen (5 μM), or N-acetyl cysteine (NAC, 1 mM). (**C**,**D**) Levels of GSH, GPX4 (**C**), 4-HNE, and MDA (**D**) in the BMDMs infected with indicated strains for 12 h. Data are shown as means ± SD of six independent biological replicates. * p < 0.05, ** p < 0.01, *** p < 0.001.

To assess whether *CVF1*-induced macrophage lysis is associated with a specific death pathway, we tested inhibitors targeting different types of cell death: z-VAD-FMK for apoptosis [34], necrostatin-1s for necroptosis [35], disulfiram for pyroptosis [36], and ferrostatin-1 (Fer-1) for ferroptosis [21,37]. The data showed that only repression of ferroptosis caused a notable reduction in LDH release (Figure 3A). Conversely, necroptosis inhibition had a minor effect on LDH activity, while blocking apoptosis or pyroptosis did not influence LDH levels (Figure 3A). Meanwhile, in vitro stress experiments showed that *CVF1* in *C. albicans* could contribute to an increased resistance to the tested oxidative stress (Figure S1D). To further explore the role of *CVF1* in ferroptosis, we used RSL3, which is a widely used inducer of ferroptosis [21]. As shown in Figure 3B, RSL3 caused a dramatic increase in *CVF1*-induced cell death in BMDMs. Furthermore, *CVF1*-induced macrophage lysis was obviously rescued by the ferroptosis inhibitors Fer-1, Ebselen, and NAC (Figure 3B). For the levels of GSH, GPX4, 4-HNE, and MDA, four markers for ferroptosis [18,38], 4-HNE and MDA were significantly elevated in WT- and complemented-strain-infected macrophages, compared with the macrophages interacting with the *cvf1* mutant (Figure 3D). However, GSH and GPX4 showed the opposite effects (Figure 3C). These data suggest that *CVF1* induces ferroptosis in *C. albicans*-infected BMDMs.

### 3.4. CVF1-Induced Ferroptosis Decreases Fungal Killing and Promotes Inflammation in Macrophages

**Figure 4:**
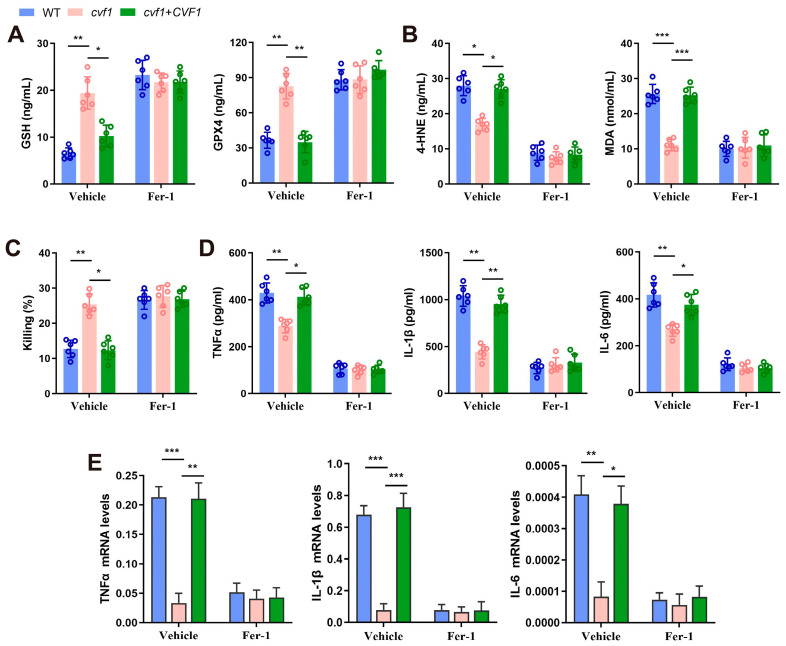
*CVF1*-induced ferroptotic cell death decreases macrophage-mediated fungal killing and promotes inflammation. BMDMs were treated with 10 μM Fer-1 for 1 hour, followed by interaction with WT, *cvf1*, or *cvf1*+*CVF1* strains (MOI 1) for 12 h. (**A**,**B**) BMDMs were infected using indicated strains with or without Fer-1, and the levels of GSH, GPX4 (**A**), 4-HNE, and MDA (**B**) were assessed. (**C**) *C. albicans* killing of macrophages in the presence and absence of Fer-1. After lysing macrophages, the viability of *C. albicans* was assessed by quantitating the number of CFUs. The percentage of killing was determined by comparison to the CFUs in the starting inoculum. (**D**,**E**) Expression levels of TNFα, IL-1β, and IL-6 were analyzed using ELISA (**D**) and qRT-PCR (**E**) from macrophages in the presence and absence of Fer-1 during infection with indicated *C. albicans* strains. qRT-PCR results were normalized to GAPDH. Error bars are means ± SD of six independent biological replicates. * p < 0.05, ** p < 0.01, *** p < 0.001.

To further confirm *CVF1*-induced ferroptosis and its effect on fungal survival and inflammation in *C. albicans*-infected macrophages, the related indicators of BMDMs infected with the *cvf1* mutant and control strains were detected. Compared with the macrophages infected with the *cvf1* mutant, those infected with the WT or complemented strain displayed decreased expression of GSH and GPX4 and elevated levels of 4-HNE and MDA. These differences were reduced upon treatment with Fer-1 (Figure 4A,B). Similar results were also found in the processing of NAC (Figure S3). These results further confirm that *CVF1* triggers ferroptosis in *C. albicans*-infected BMDMs.

Next, the effect of ferroptosis induced by *CVF1* on *C. albicans* survival in the BMDMs was determined. As shown in Figure 4C, the WT or complemented strain interacting with macrophages exhibited significantly higher fungal survival than the *cvf1* mutant strain interacting with macrophages. Fer-1 treatment abolished this phenomenon. This finding suggests that the survival of *C. albicans* aided by *CVF1* is modulated via the induction of ferroptosis in macrophages.

Compelling evidence has documented that ferroptosis possesses a critical role in driving inflammation during *C. albicans* infections [21]. We investigated whether *CVF1*-induced ferroptosis contributes to inflammatory responses in macrophages. As expected, macrophages infected with the WT or complemented strains exhibited higher levels of proinflammatory cytokines relative to those with the *cvf1* mutant strain. This increase in cytokine levels was reversed by treatment with Fer-1 (Figure 4D,E). Taken together, *CVF1* triggers both macrophage death and an inflammatory response via activating ferroptosis.

### 3.5. CVF1 Induces Ferroptosis to Enhance C. albicans Pathogenicity and Dissemination In Vivo

**Figure 5:**
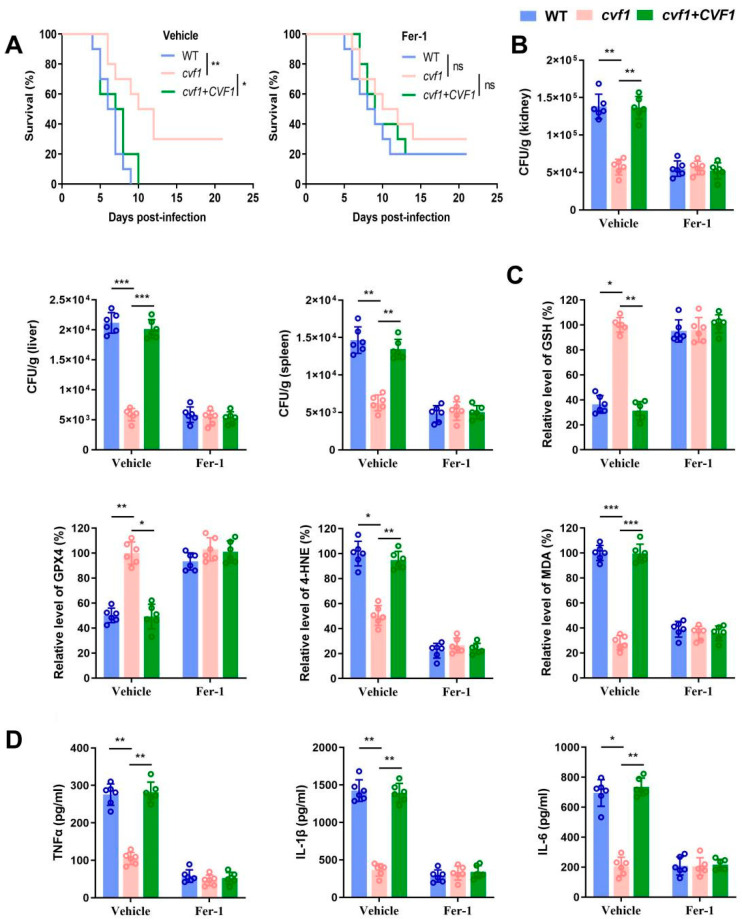
*CVF1* induces ferroptosis to promote *C. albicans* pathogenicity and dissemination in vivo. Male BALB/c mice were infected with 5 × 10^5^ CFU WT, *cvf1*, or *cvf1*+*CVF1* strains via their lateral tail vein and were then treated intraperitoneally with 10 mg/kg of Fer-1 or the vehicle control daily. (**A**) Survival of BALB/c mice infected with the indicated strains was monitored. Statistical significance was determined using the Mantel–Cox test. (**B**) The fungal burden in the tissues (kidneys, liver, and spleen) of BALB/c mice infected with indicated *C. albicans* strains at day 5. (**C**) The levels of GSH, GPX4, 4-HNE, and MDA in homogenized kidneys from BALB/c mice infected with indicated *C. albicans* at day 5. The mean of the WT vehicle control group was set at 100% to determine the percentage change of other groups. (**D**) The levels of TNFα, IL-1β, and IL-6 in homogenized kidneys from BALB/c mice infected with indicated *C. albicans* at day 5. Data represent the means ± SD of six independent biological replicates. ns, not significant; * p < 0.05, ** p < 0.01, *** p < 0.001.

Ferroptotic cell death plays a critical role in the pathogenicity of disseminated candidiasis [21]. To elucidate the role of *CVF1*-induced ferroptosis in *C. albicans* pathogenicity and dissemination, we examined the effects in mice treated with Fer-1. Mice infected with the WT or complemented strains exhibited lower survival rates (Figure 5A and Figure S4) and notably higher fungal loads in the kidneys, liver, and spleen relative to those infected with the *cvf1* mutant strain (Figure 5B). In line with our in vitro findings, mice infected with WT or complemented strains exhibited elevated levels of 4-HNE and MDA, reduced expression of GSH and GPX4 (Figure 5C), and elevated expression of proinflammatory cytokines in their kidneys (Figure 5D) compared with mice infected with the *CVF1* mutant strain. Meanwhile, Fer-1 treatment could abolish the above differences between mice infected with WT or complemented strains and mice infected with the *cvf1* mutant strain. Collectively, *CVF1*-induced ferroptosis promotes inflammation and *C. albicans* pathogenicity during systemic infection.

## 4. Discussion

Regulated cell death is critical in shaping interactions between pathogens and their hosts [39]. Ferroptosis, a relatively recent addition to the known forms of regulated cell death [11], has been implicated in pathogen survival strategies. Emerging evidence suggests that some pathogenic bacteria and fungi exploit host ferroptosis to secure their replicative niches within the host [13,15]. However, the specific virulence factors of pathogens that regulate ferroptosis to promote infection remain poorly understood [11,18]. In the case of human pathogenic fungi, it is still uncertain whether specific virulence factors trigger ferroptosis in host cells to enhance colonization and virulence. *C. albicans*, a most common human pathogenic fungus causing mucosal infections and systemic infection [40], has recently been found to be able to trigger ferroptosis in macrophages to promote systemic infection [21]. However, the virulence factor of *C. albicans*-induced ferroptosis in host cells is still unknown.

Identifying virulence factors is essential for comprehending the molecular mechanisms through which pathogens cause diseases [41]. The genome of *C. albicans* encodes over 6200 genes, yet the functions of only 22.97% of these genes have been experimentally confirmed. A large portion of the genome, roughly 4700 genes, remains uncharacterized, as documented in the CGD database (www.candidagenome.org) and related studies [42,43]. Among these genes of *C. albicans* with unknown functions, there may be potential key virulence genes. In fact, researchers in the field of *Candida* have been trying to identify the virulence factors of *C. albicans*, such as the recent identification of two key virulence genes during systemic infection, *LIP2* [44] and *CMI1* [33]. Our findings reveal that *CVF1* is a key virulence gene during systemic infection, and this gene significantly promotes macrophage death and inflammatory responses. *CVF1* has homologous genes in other *Candida* pathogenic fungi (including *Candida dubliniensis* and *Candida tropicalis*) (http://www.candidagenome.org/), but the prevalence of these *Candida* species varies [45]. It may be the case that the *CVF1* of these *Candida* species can be retained through a special evolutionary mechanism and play a similar role in the process of their interaction with the host. For example, *CVF1* may play a key role in the lysis of macrophages induced by these *Candida* species or promote the transformation of these *Candida* species from symbiotic fungi to pathogenic fungi. These hypotheses need further study. Meanwhile, because *C. albicans* is only a branch of pathogenic fungi, even among phylogenetically closely related *Candida* species (e.g., *C. dubliniensis*), their prevalence and threat to patients are very different [45]. Therefore, whether all human pathogenic fungi can induce ferroptosis in macrophages and whether the key virulence factors causing ferroptosis in host cells have homology remain to be further investigated via experimental approaches. The discovery of *CVF1* as a critical virulence gene in *C. albicans* infections offers a potential target for developing new strategies to prevent and manage candidiasis while also opening avenues for further investigation into the role of *CVF1* in macrophage-regulated cell death.

Recently, a few studies have identified virulence genes that trigger host cell ferroptosis in pathogenic bacteria. For example, the PtpA effector protein of *M. tuberculosis* can induce ferroptosis in host cells to enhance pathogenicity [18]. In addition, one gene of *P. aeruginosa* has been found to cause macrophage ferroptosis and lysis [46]. At present, most of the virulence genes identified in human pathogenic fungi directly regulate the host immune system, and those that regulate the programmed cell death of infected host cells are very limited. For example, Dang EV et al. [47] have documented *C. neoformans CPL1* as a key virulence gene potentiating IL-4 signaling via TLR4 in macrophages. Meanwhile, we have recently found that the novel virulence gene *CMI1* of *C. albicans* can block type I interferon signaling in host cells [33]. In this study, our findings revealed that *CVF1* significantly promoted macrophage lysis and *C. albicans* pathogenicity in mice by triggering ferroptosis. Our research confirms the presence of a specific virulence factor in human pathogenic fungi that mediates host cell ferroptosis.

In sum, we provide the first report, to our knowledge, of pathogen-specific virulence factors activating host cell ferroptosis in the context of interactions between human pathogenic fungi and hosts. We identify *CVF1* as a critical virulence gene of *C. albicans* that promotes fungal virulence by inducing ferroptosis in macrophages. Additionally, *CVF1* is necessary for virulence in vivo, promoting ferroptosis during systemic infections in mice. We postulate that the strategy for targeting *CVF1* may be another means to reduce invasive candidiasis. Future research will be essential to (i) elucidate the role of *CVF1* in mucocutaneous infections, (ii) understand the molecular mechanism of ferroptosis activation by *CVF1*, and (iii) confirm the presence of ferroptosis in other human pathogenic fungal infections and understand its mechanisms.

## Data Availability

The original contributions presented in this study are included in the article/Supplementary Materials. Further inquiries can be directed to the corresponding authors.

## Supplementary Materials

The following supporting information can be downloaded at: https://www.mdpi.com/article/10.3390/jof11050342/s1, Figure S1. The effect of *CVF1* on the hyphal development and growth of *C. albicans* under in vitro conditions. (A, B) *CVF1* is not involved in the morphology of *C. albicans* under in vitro liquid hypha-inducing conditions. WT, *cvf1*, or *cvf1*+*CVF1* strains were incubated in YPD+10% serum (A), or RPMI (B) for 0-5 h at 37 °C, then cells were monitored. (C) WT, *cvf1*, or *cvf1*+*CVF1* strains were cultured in YPD medium at 30 °C, and the OD_600_ value was measured at indicated time points. (D) Deletion of *CVF1* increases sensitivity to oxidative stress conditions. The WT, *cvf1*, or *cvf1+CVF1* strains were grown in YPD at 30 °C until the exponential phase, and then exposed to different concentrations (0, 2.5, 5, 10, 20, 50 mM) of H_2_O_2_ at 30 °C for 60 min (left), were also treated with different NaNO_2_ concentrations (0, 2.5, 5, 10, 20, 30 mM) at 30 °C for 4 h (right). Data represent the mean ± SD of at least three independent biological replicates. *** p* < 0.01, **** p* < 0.001. Figure S2. Bioinformatics analysis of Cvf1 in *C. albicans*. (A-C) Schematic diagram of structural domain analysis (A), functional prediction (B), and the STRING (version 12.0) protein-protein interaction network (C) of Cvf1. NLS refers to nuclear localization signal. Structural domain analysis is mainly conducted through PredictProtein and PSORT online software, while functional prediction is performed using FFPred online program (version 4). After importing Cvf1 amino acid sequence, the corresponding software’s default parameters are used for analysis. Figure S3. (A-D) After BMDMs were treated with NAC (1 mM) for 1 hour followed by interaction with WT, *cvf1*, or *cvf1*+*CVF1* strain (MOI 1) for 12 h, the levels of GSH (A), GPX4 (B), 4-HNE (C) and MDA (D) were detected. Error bars are means ± SD of six independent biological replicates. ** p* < 0.05, *** p* < 0.01. Figure S4. Survival of female BALB/c mice infected with the indicated strains. Female BALB/c mice were infected with 5×10^5^ CFU WT, *cvf1*, or *cvf1*+*CVF1* strain via lateral tail vein, then were treated daily intraperitoneally with 10 mg/kg Fer-1 or vehicle control. Mantel-Cox test was used for statistical significance. ns, not significant; ** p* < 0.05, *** p* < 0.01. Table S1 Strains used in this study. Table S2 Primers used in this study. Reference [22] is cited in Supplementary Materials.
