# Supplementary material for: CVF1 Promotes Invasive Candida albicans Infection via Inducing Ferroptosis
Source: J Fungi (Basel). 2025 Apr 27;11(5):342. doi: 10.3390/jof11050342 (PMC12113316; doi:10.3390/jof11050342)
Supplement: Supplementary file 1 [file jof-11-00342-s001.zip › Table S1 Strains used in this study.pdf]

Table S1. Strains used in this study

Strain	Relevant genotype	Source or reference
SN152	*arg4Δ/arg4Δ leu2Δ/leu2Δ his1Δ/his1Δ URA3/ura3Δ::imm*^{434} *IRO1/iro1Δ::imm*^{434}	(1)
cvf1Δ/Δ	*cvf1Δ::HIS1/ cvf1Δ::LEU2 arg4Δ/arg4Δ URA3/ura3Δ::imm*^{434} *IRO1/iro1Δ::imm*^{434}	This study
cvf1Δ/Δ::CVF1	*cvf1Δ::HIS1/ cvf1Δ::CVF1::ARG4 URA3/ura3Δ::imm*^{434} *IRO1/iro1Δ::imm*^{434}	This study

References

1. Noble SM & Johnson AD (2005) Strains and strategies for large-scale gene deletion studies of the diploid human fungal pathogen *Candida albicans*. *Eukaryotic cell* 4(2):298-309.
