# Supplementary material for: CVF1 Promotes Invasive Candida albicans Infection via Inducing Ferroptosis
Source: J Fungi (Basel). 2025 Apr 27;11(5):342. doi: 10.3390/jof11050342 (PMC12113316; doi:10.3390/jof11050342)
Supplement: Supplementary file 1 [file jof-11-00342-s001.zip › Table S2 Primers used in this study.pdf]

Table S2. Primers used in this study

Primer	Sequence
CVF1 P1 / R1	CTCCTCAATTTATTGACATTTAATTTTC
CVF1 P3	CACGGCGCGGCTAGGAGCGGCGGGAAGTTAATTGATGGTTC
CVF1 P4 / R3	CTCAGCGGCCGCATCGCTGCGATTTTACAATATTCCACAGAAGTTAC
CVF1 P6	AATTGATATATTTGGTGAAGAACTAGC
CVF1 R2	CACGGCGCGGCTAGGAGCGGGCTGTAGGTAGTTTAATTTGTAGTAAAT
CVF1 R4	CCGTATAATGGTTCGATATTGAG
CVF1 Check 1 F	CCATTTGTCTTCCTTTCTTTTTATTG
CVF1 Check 1 R	ATGCTGTAGGTAGTTTAATTTGTAG
CVF1 Check 2 F	GTCTTCAACAAATAGTATCTATGATCC
CVF1 Check 2 R	TATGGAGATTGACGGTATATCTG
