# Supplementary figures and images for: CVF1 Promotes Invasive Candida albicans Infection via Inducing Ferroptosis
Source: J Fungi (Basel). 2025 Apr 27;11(5):342. doi: 10.3390/jof11050342 (PMC12113316; doi:10.3390/jof11050342)

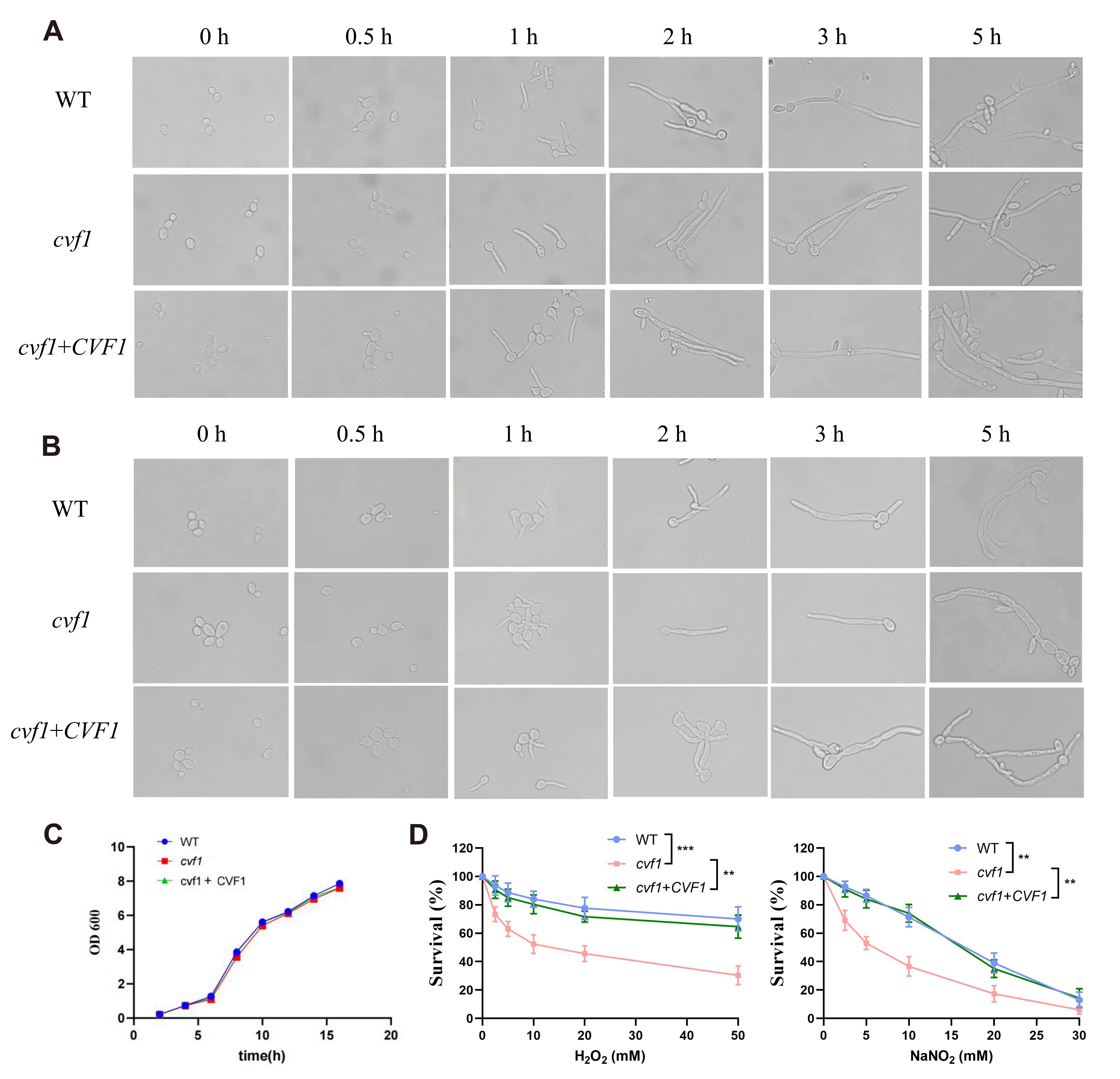

Supplement: Supplementary file 1 [file jof-11-00342-s001.zip › Figure S1.jpg]

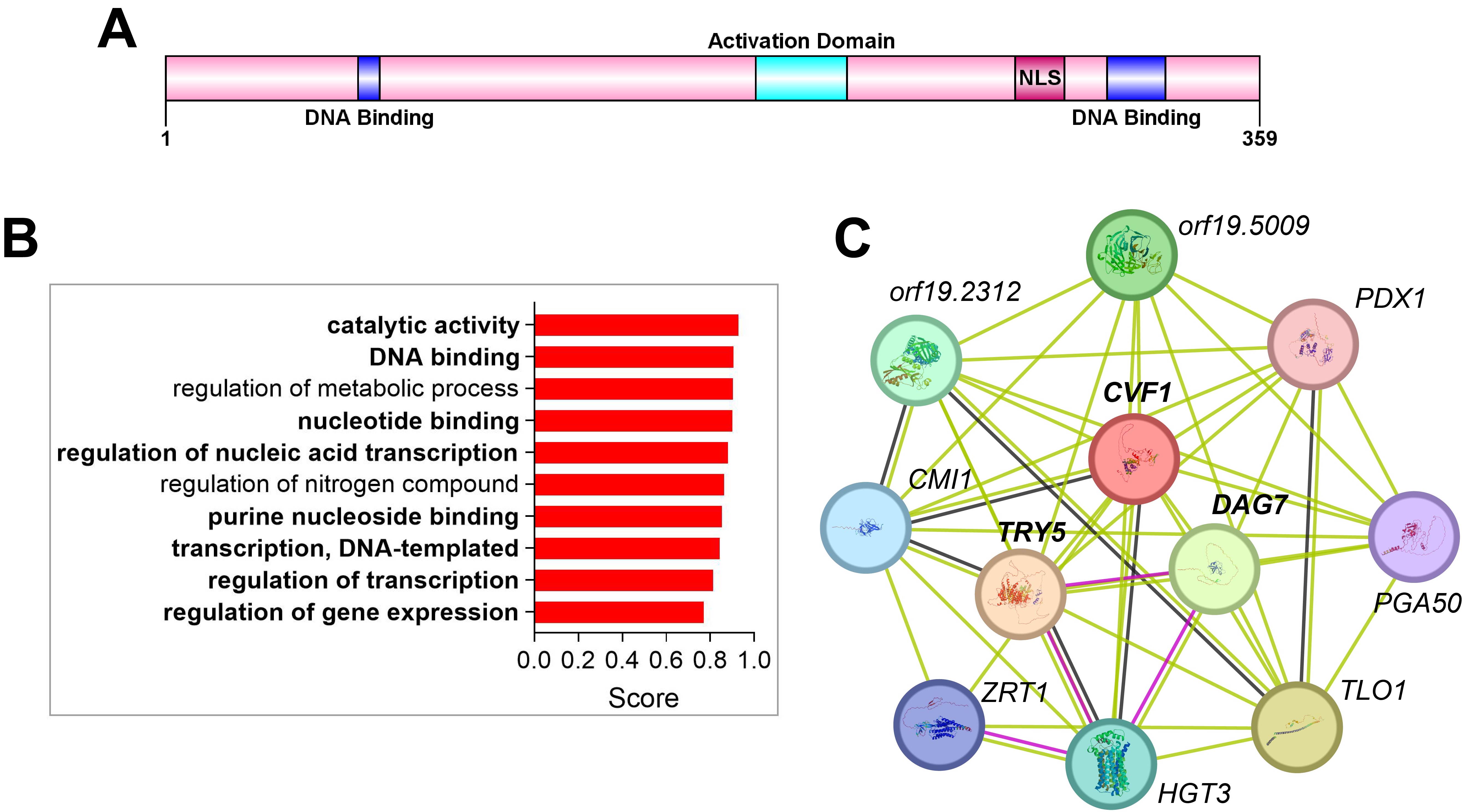

Supplement: Supplementary file 1 [file jof-11-00342-s001.zip › Figure S2.jpg]

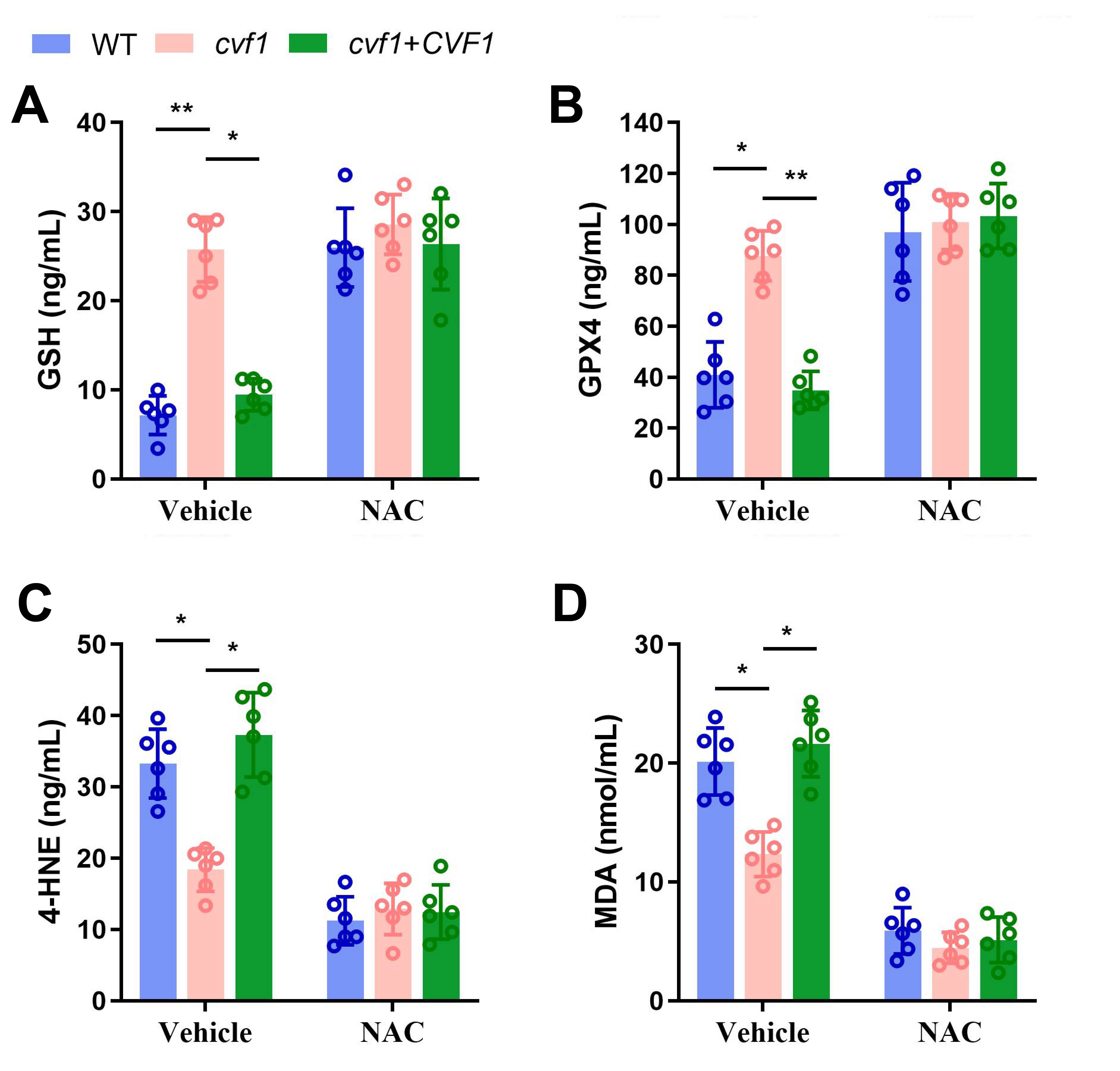

Supplement: Supplementary file 1 [file jof-11-00342-s001.zip › Figure S3.jpg]

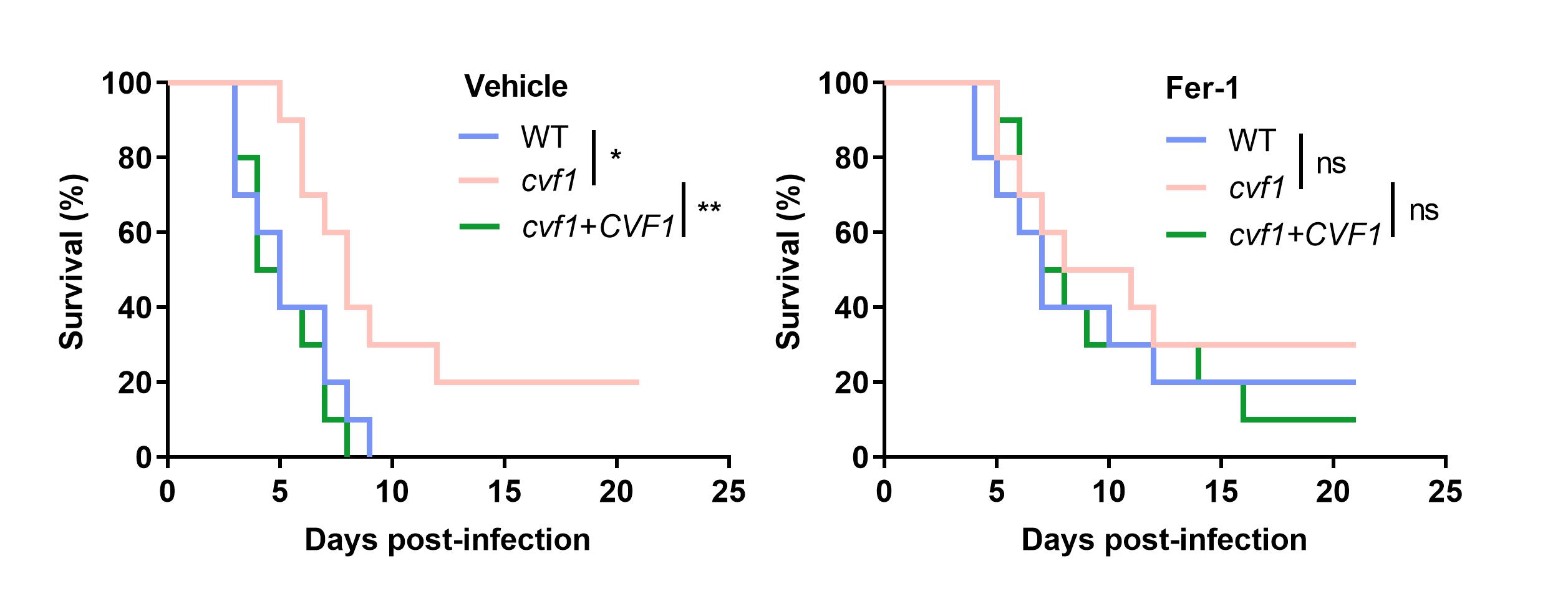

Supplement: Supplementary file 1 [file jof-11-00342-s001.zip › Figure S4.jpg]
